# A technical review and evaluation of implantable sensors for hearing devices

**DOI:** 10.1186/s12938-018-0454-z

**Published:** 2018-02-13

**Authors:** Diego Calero, Stephan Paul, André Gesing, Fabio Alves, Júlio A. Cordioli

**Affiliations:** 1 Laboratory of Vibration and Acoustics, Florianópolis, Brazil; 2 Naval Postgraduate School, Monterey, CA USA

**Keywords:** Cochlear implant, Hearing aids, Implantable transducers, Subcutaneous microphone, Piezoelectric sensor, Accelerometer, MEMS sensor

## Abstract

Most commercially available cochlear implants and hearing aids use microphones as sensors for capturing the external sound field. These microphones are in general located in an external element, which is also responsible for processing the sound signal. However, the presence of the external element is the cause of several problems such as discomfort, impossibility of being used during physical activities and sleeping, and social stigma. These limitations have driven studies with the goal of developing totally implantable hearing devices, and the design of an implantable sensor has been one of the main challenges to be overcome. Different designs of implantable sensors can be found in the literature and in some commercial implantable hearing aids, including different transduction mechanisms (capacitive, piezoelectric, electromagnetic, etc), configurations microphones, accelerometers, force sensor, etc) and locations (subcutaneous or middle ear). In this work, a detailed technical review of such designs is presented and a general classification is proposed. The technical characteristics of each sensors are presented and discussed in view of the main requirements for an implantable sensor for hearing devices, including sensitivity, internal noise, frequency bandwidth and energy consumption. The feasibility of implantation of each sensor is also evaluated and compared.

## Background

Several forms of hearing loss can be treated through the use of hearing devices, such as hearing aids (HAs), middle ear implants (MEIs) or cochlear implants (CIs). With few exemptions, these devices use one or more microphones, located in a behind-the-ear device or in the outer ear canal, to capture the sound field, which can be in turn processed and transmitted forward. In HAs, the sound is processed, amplified and sent into the ear canal using vibro-acoustic systems (speakers^1^). In MEIs the signal is transformed into vibration to stimulate the ossicular chain. Both approaches are appropriate for mild to moderate conductive hearing loss, however, not effective in the case of severe or profound sensorineural hearing loss. In these cases, the CI—which uses an electrode array inserted into the cochlea to stimulate the auditory nerve fibers—appears as an alternative. In currently available CIs, the sound signal is also picked up by microphones located in a behind-the-ear unit and it is then processed and transmitted via radio frequency (RF) to a subcutaneous element surgically implanted on the temporal bone. This internal element is responsible for generating a corresponding current pulse train, which is sent to an electrode array implanted in the cochlea, usually through the round window, resulting in a direct electric stimulation of the auditory nerve fibers.

All types of hearing devices considerably improve the life of millions of people suffering of hearing loss around the globe [1, 2]. However, these devices still display several limitations, and considerable efforts have been made to further improve such technologies. One key aspect that imposes limitations to their use is the presence of a external elements, which houses the microphones, signal processor, battery and, in the case of MEIs and CIs, the RF antenna. Besides the cosmetic aspect, patients relate that the existence of external parts of the device imposes drawbacks such as: vulnerability of the device, the possibility of detachment , to break and to be lost or stolen [1, 3]. Further on, most devices cannot be used under water, during intense physical activities or even while the user is asleep. Wind noise is also related as a inconvenience issue, and users also might have difficulties wearing helmets. And, at last, the magnetic fixation of the RF antenna can cause problems in the skin tissue due to continuous pressure exercised by the antenna. The wish to overcome these limitations has driven the development of totally implantable hearing aids (TIHAs), sometimes also called totally implantable middle ear implants and totally implantable cochlear implants (TICIs) [4, 5].

The design of an implantable sensor, which displays similar performance to the traditional electret microphones [6, 7], has been one of the challenges to be overcame in the development of TIHAs and TICIs. In the last years, different solutions have been proposed, considering different transduction mechanisms and locations for the implantable sensor. For example, subcutaneous microphones have been proposed for TIHAs and TICIs since 2000 [8–10], and are currently found in the commercial *Carina* device [11]. Another group of solutions comprises sensors implanted in the middle ear (ME), operating as microphones [12], accelerometers [13] or force sensors [14], with the latter being currently used in the commercially available TIHA *Esteem*  [15]. Efforts have also been made to reduce sensor size by using MEMS (microelectromechanical systems) technology, by applying several different transduction principles including piezoresistivity [16, 17], piezoelectricity [18, 19] and the capacitive effect [20–22].

The performance of most of the implantable sensors has been analyzed by means of different techniques, including lumped parameter models [23], finite element (FE) models [19], or experimentally through tests with prototypes in laboratory set-ups [24] or directly in animal or human temporal bones (TBs) [16, 20]. Nevertheless, for the sensor designs that can be found in TIHAs currently commercialized (*Carina* and *Esteem*) technical information is quite scarce, and the literature related to these commercial devices [25, 26] have focused mainly on patient satisfaction and clinical evaluation. Although a recent review on implantable sensors for hearing devices has been presented [27], a thorough technical comparison between different approaches is still inexistent; i.e. available information on technical characteristics and performance of implantable sensors is either scattered or incomplete.

The present work aims to review the information available on implantable sensors and attempts to standardize the presentation of the parameters used to compare their performance. To this extent, the present article starts with a discussion of the general requirements for implantable sensors for hearing devices, including bandwidth, dynamic range, sensitivity, internal noise and power consumption. From the analysis of sensors proposed in the literature or sensors currently in use by commercial devices, a general classification scheme is proposed, based on the location of implantation, transduction mechanisms and configurations. The technical characteristics of each sensor are presented, and some procedures are proposed in order to obtain data that is not directly available in the literature. Finally, the performance of the sensors and their implantation feasibility is compared with regards to the main requirements and information obtained from the literature. It is expected that this compilation of data will guide the selection of sensor technology for future hearing devices.

## Hearing devices general sensor requirements and sensor performance

The main requirements for an implantable sensor are defined by the characteristics of human hearing, mainly dynamic range and frequency range, and the characteristics of the input sound, focused particularly in speech signals. Frequency range of human hearing is commonly stated to extend from 20 Hz to 20 kHz [28], whereas human speech is mostly constrained from 250 Hz to 4 kHz. Within the frequency range of human hearing, the auditory system is most sensitive between 2 and 4 kHz [29].

In general, a sensor for a hearing device requires a broad frequency response, but not extending to very low frequencies (not below 200 Hz), in order to minimize the response to vibrations produced by body movements [30]. Capturing high frequency sounds (between 4 and 8 kHz) is important for speech understanding, particularly in noisy situations [31]. In line with these requirements most of the microphones currently used in conventional HAs and CIs have a frequency range from 100 Hz to 8 kHz [32], and for implantable sensors, a bandwidth from 250 Hz to 8 kHz has been proposed [20]. Nevertheless, taking into account that several important environmental sounds are at lower frequencies (below 200 Hz), the lower frequency limit has been defined to be 100  [18], but it should be noted that there exists an overlap of environmental and body sounds between 100 and 200 Hz and this overlap can be an intrinsic limitation of the implantable sensor. Concluding, ideally the frequency range would therefore comprise frequencies from around 100 Hz to 8 kHz and frequency response should be flat within this range in order to avoid the use of compensation filters and therefore reduce DSP power consumption.

The dynamic range of human hearing is function of the frequency, since the threshold of hearing as well as the threshold of discomfort or pain vary with the frequency. The threshold of discomfort varies between 80 and 100 dB sound pressure level (SPL) [33] in the hearing frequency range, whereas the hearing threshold is minimum, and even as low as − 5 dB, between 3 and 4 kHz [29]. Useful dynamic range of the auditory system, taking into account that the upper limit is the threshold of discomfort, varies between 80 and 100 dB, and is largest at 3–4 kHz. Compared to this useful dynamic range of the human hearing system the dynamic range of human speech is 60 dB for English [34].

Most current hearing devices use microphones that are sensitive to sound pressure levels ranging from around 30 to 140 dB SPL [35], which matches this dynamic range. Considering that the sensitivity of human hearing is a function of frequency, the input range has also been defined from 30 to 100 phon [23], what equals 30 and 100 dB SPL at 1 kHz respectively. In general, the lower limit of the sensor’s dynamic range is defined by the sensor’s internal noise, while the superior limit marks the beginning of significant distortions/non-linearities of the sensor. For sensors implanted in the middle ear, an appropriate dynamic range has been assumed to be from 40 to 100 dB SPL applied to the tympanic membrane. Additionally it has been suggested that the sensor must be designed for sudden amplitude changes, produced by shocks or air pressure variations [20].

**Fig. 1:**
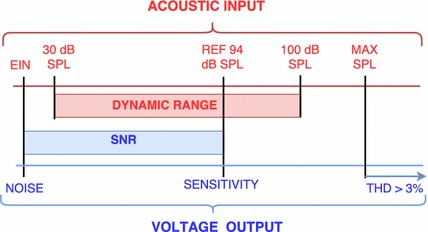
Ranges relation between acoustic input and voltage output ranges

The requirements related to sensitivity and output noise of the sensor depend mainly on the dynamic range previously specified, as commonly recommended for microphones and compared in Fig. 1 [35, 36]. The sensor sensitivity is obtained for a reference input sound pressure of 1 Pa (94 dB SPL), and in the case of sensors implanted in the ME, it is measured in the ear canal near the tympanic membrane. The equivalent input noise (EIN) is the difference between the reference input (94 dB SPL) and the signal to noise ratio (SNR), and must be lower than the inferior bound of the previously specified dynamic range.

The maximum accurately measurable SPL—where the response of the sensor becomes nonlinear or the total harmonic distortion (THD) reaches a specified amount, typically 3% for microphones  [37]—must be higher than the dynamic range upper bound, which has been defined previously as 100 dB SPL.

Sensor’s EIN and sensitivity greatly vary according to the transduction principle. For example, sensitivity of common electret condenser microphones (ECM), with a 2.5 mm diameter membrane, used in conventional hearing aids, varies from 20 to 30 dB ref 1 mV/Pa [6, 35, 38]. Whereas the sensitivity of a piezoresistive MEMS accelerometer has been reported as mere 6 dB [16, 39], and for capacitive sensors varied from − 9 to 30 dB [20, 23, 40]. Similarly, ECM’s EIN may be as low as 20 dB SPL at 1 kHz [7], whereas a capacitive accelerometer implanted in the middle ear may detect only SPL above 35 dB at the same frequency [21], and yet a piezoresistive accelerometer’s EIN may be 60 dB SPL at 1 kHz [16].

Typically, piezoelectric MEMS microphones have had a much higher equivalent noise level than their ECM counterparts, but more recently EIN has been reduced to 30 dBA for single piezoelectric MEMS sensors [41] and to 27 dBA for multiple sensor arrangements [7].

Non acoustical requirements for implantable sensors include bio-compatibility, sealing, size and mass limitations and power consumption. In the case of a sensor coupled to the ME, its size must allow for its handling and implantation in the limited space of the ME cavity. Sachse et al. [23] suggests a maximum dimension of 2 mm for middle ear sensors with a single point of attachment as in the accelerometer configuration. In the case of sensors which also considers a fixation point to ME cavity walls, such as the *Esteem* device [15], the limitations to the sensor size are less restrictive and are likely based on the available space within the ME cavity. If the implantable sensor operates in the an accelerometer configuration, its mass must also be restricted in order to avoid changing the dynamics of the middle ear, what may affect the patient’s residual hearing [20]. In this case, Ko et al. [20] recommends the mass to be less than 10% of the ossicle’s mass to which it will be coupled (malleus 23–35 mg, incus 25–38 mg, stapes 2–4.5 mg [42–44]). In this sense, electrical wire mass and stiffness should also be taken into account [16]. In the case of a sensor operating as force transducer, its stiffness must be taken into account as well, and a analysis of the dynamic response of the ossicular chain must be carried out [18, 45].

Energy consumption is another important aspect for implantable sensors since battery life will be a key factor for any implantable hearing device. Therefore, the power required by an implantable sensor should be minimized. It is important to mention that the power consumption depends on the amplifier–sensor combination, since both affect the overall noise and power consumption of the sensor [41]. Typically, ECMs and their amplifying electronics for traditional hearing devices consume around 0.05–0.5 mW [6, 46], which is between 1 and 10% of the power provided by the battery for the entire device.^2^ While Ko et al. [20] recommend that the energy consumption of an implantable sensors should be less than 1 mW, a similar energy consumption observed in ECMs should be reached, considering battery life and its charging or replacement specifications for the entire hearing device.

## Description of implantable sensors

**Fig. 2:**
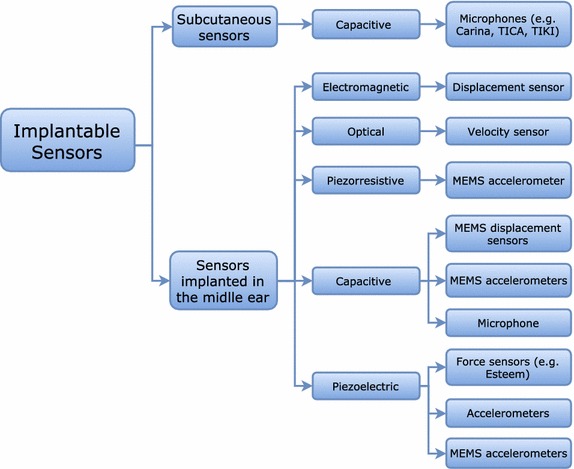
Classification of implantable sensors available in the literature for hearing devices on three levels: positioning, transduction mechanism and sensor type

Based on an estensive literature review, a classification scheme of available technologies for implantable sensors is suggested in Fig. 2. This classification is based, primarily on the sensor positioning: subcutaneous or implanted in the ME ossicular chain, Secondly, the classification considers the transduction mechanism: capacitive, electromagnetic, optical, piezoresistive and piezoelectric. Finally, the classification accounts for the sensor type: microphone, accelerometer, displacement sensor and force transducer.

Implantable sensors described in the literature are listed in Table 1 alongside the most significant references, their research status and evaluation methods used to assess sensor performance. An alphanumerical code was added to identify the sensors and will be used throughout the text, where a capital letter represents the sensor type, and a number for each specific example is added. Each sensor is described in this section, and their performance is analyzed and compared in the following section.

**Table 1:** Summary of the designs proposed in the literature for implantable sensors. An alphanumerical system is added to better comparison, and the status of each study is briefly described

Type	Name/reference, year	ID	Status
Subcutaneous microphones (A)	*TICA*: Zenner et al. 2001 [9]	A1	TIHA, not sold anymore, initially developed by *Implex*, 20 patients reported
	*TIKI*: Briggs et al. 2008 [10]	A2	TICI prototype, 3 patients reported
	*Carina*: Jenkins et al. 2007 [11, 48]	A3	TIHA commercialized by *Otologics* (now *Cochlear*), 110 patients reported
	Jung et al. 2011 [49], Jung et al. 2012 [50]	A4	Prototype tested with artificial skin
Electromagnetic sensor (B)	Maniglia et al. 2001 [51]	B	Prototype tested in laboratory bench and TBs
Optical sensor (C)	Vujanic et al. 2002 [52]	C	Prototype tested with a piezoelectric shaker
Piezoresistive MEMS accelerometer (D)	Park et al. 2007 [16]	D	Prototype tested in TBs
Capacitive MEMS displacement sensor (E)	Huang et al. 2007 [22]	E1	Prototype tested in TBs
	Ko et al. 2009 [20]	E2	Prototype tested in TBs
Capacitive MEMS accelerometer (F)	Zurcher et al. 2007 [21], Ko et al. 2009 [20], Young et al. 2012 [40]	F1	Prototype tested on a laboratory bench and using TBs
	Sachse et al. 2013 [23]	F2	Lumped parameter model and prototype tested in TBs
Capacitive microphone (G)	Woo et al. 2012 [53], Woo et al. 2013 [12]	G	Prototype tested in animals
Piezoelectric force sensor (H)	Javel et al. 2003 [14]	H1	Prototype tested in animals
	*Esteem*: Chen et al. 2004 [15, 54]	H2	TIHA commercialized by *Envoy Medical*, 134 patients reported
	Koch et al. 2013 [55], Koch et al. 2014 [24]	H3	FE model and prototype tested in laboratory and TBs
Piezoelectric accelerometer (I)	Kang et al. 2012 [13], Gao et al. 2013 [56], Jia et al. 2016 [56]	I	FE model and prototype tested in animals and TBs
Piezoelectric MEMS accelerometer (J)	Beker et al. 2013 [19]	J1	FE model and prototype tested in laboratory
	Yip et al. 2015 [18]	J2	Lumped parameter model and prototype tested in TBs

### Subcutaneous microphone

The conventional capacitive microphone comprises a diaphragm that, when deformed by sound pressure, generates an electrical signal through a capacitive mechanism. Capacitive sensing for microphones usually works by measuring changes in capacitance between two conductive plates, or a backplate and a membrane, when a voltage difference is applied to them.

The most commonly encountered capacitive microphone is the ECM, which uses a material which is permanently polarized, called electret. Also, all commercially available MEMS microphones in 2015 use capacitive means of detection [57, 58], featuring a rigid backplate and flexible membrane that deflects out of the wafer plane. Downscaling capacitive microphones is problematic, as sensitivity depends on capacitance. Moreover capacitive sensors are highly sensitive to parasitic capacitance and nonlinearity [57, 58].

Implantable microphones for hearing devices have the same working principle as ECM, with the main difference that the acoustic wave will need to cross a layer of tissue before reaching the diaphragm. Most implantable microphones have been placed under the skin of the head, either directly above the pinna or in the bony walls of the ear canal. Implantation above the pinna has been the preferred option [30], enabling the use of a larger area for the diaphragm, and even allowing to place a microphone arrangement in order to increase the directional selectivity or to benefit from increased SNR [7]. Nevertheless, the first TIHA, called *TICA* (A1), and commercialized by *Implex* in 2001 [9], used a subcutaneous microphone implanted under the skin of the ear canal, with the justification that by making use of the natural acoustical resonance of the ear canal, the overall performance could be improved. The hermetically sealed microphone consisted of a titanium cylinder with a 4.5 mm diameter titanium membrane weighting 0.4 g. The *TICA* was implanted in 20 patients [9], and there were no further studies reported with patients using this device since 2001.

The first TICI prototype was called *TIKI* (A2), or invisible CI, and also used capacitive microphone technology for sensing. The device was developed by *Cochlear* and the University of Melbourne, and is described in [10]. The device included both a subcutaneous microphone and an external microphone from the external element of a conventional CI, which worked simultaneously. The sensing mode could be changed with an external control. The prototype subcutaneous electret microphone was encapsulated together with the processor and the lithium-ion battery in a single housing 7.5 mm × 28 mm × 28 mm, which makes it larger than the conventional CI package. TIKI was intended to be implanted under the skin and the procedure was carried on in three patients with severe to profound sensorineural hearing loss. The response of the subcutaneous microphone was measured in the implanted patients, while the performance of the entire device in terms of functional gain and word recognition was compared to the same aspects in users of conventional CIs.

**Fig. 3:**
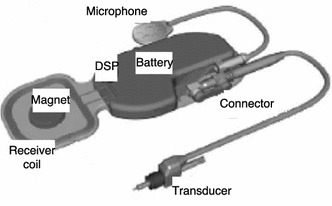
Carina parts of TIHA *Carina* (Adapted from [48], available from PubMed Central)

**Fig. 4:**
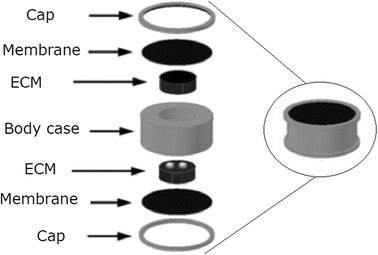
Subcutaneous microphones scheme of combined subcutaneous microphones (Adapted from [61], available from Korea Institute of Science and Technology Information)

The TIHA *Carina* (A3) is the fourth generation of a TIHA commercialized by the company *Otologics* (now *Cochlear*). As can be seen in Fig. 3, the device consists of an implantable unit comprising the sensor, the battery, and the actuator (named Transducer in Fig. 3) to be coupled to the incus or the stapes. Detailed technical characteristics of the device have not been described in the literature, but some reviews [25, 26] state that *Carina* has two ECMs, one of them oriented to the outside to capture external sounds, and the other to the inside to capture body signals, allowing body noises to be then canceled out by the DSP. This configuration has also been mentioned in some patents [59, 60]. A possible arrangement of two ECMs into a single unit, presented in a subsequent study [61], is shown in Fig. 4. Three suitable locations were considered for the subcutaneous microphone unit: retro-auricular, top of the mastoid bone, and above the pinna. The microphone position was found to be crucial for the optimal functioning of the device due to the variations in tissue thickness [11]. While technical details are not available to the public, *Carina* received the European Union CE Mark in 2007 and clinical studies were conducted for FDA approval in the United States [2, 48]. A total of 110 patients got the device implanted and evaluated from the clinical point of view [25].

Another subcutaneous capacitive microphone prototype (A4) has been studied as an alternative TIHA sensor by Jung et al. [49, 50]. The device consists of a titanium membrane (diameter 12 mm), including an acoustic tube made of titanium, in order to increase the first natural frequency. The prototype’s frequency response was measured using artificial skin made of silicon. No further tests with the prototype (A4) implanted in patients have been reported.

### Electromagnetic sensors

One of the first sensors implanted in the ME was an electromagnetic displacement sensor prototype (B) presented by Maniglia et al. in 2001 [51] as an implantable sensor for a TICI. The 29 mg displacement sensor consists of a small titanium encapsulated neodymium–iron–boron magnet glued to the head of the malleus. The magnet interacts with an electric coil placed on a titanium shaft supported in the TB at a distance of 0.5 to 1 mm from the magnet. Its prototype was tested in a laboratory set-up, using a piezoelectric diaphragm simulating the tympanic membrane and ossicular chain, and in fresh human TBs. No further tests in patients have been reported.

### Optical sensors

Another, quite different, implantable sensor solution is an optical sensor (C) implanted in the ME cavity, as proposed by Vujanic et al. in 2002 [52]. A prototype was tested in a laboratory set-up, using a piezoelectric actuator to simulate the ossicles’ vibration. The device measures the vibration of the tympanic membrane (or one of the ossicles) through the reflection of a laser beam radiated by an elastic optical fiber with a diameter of 0.125 mm. The incident and reflected beams are captured by two photo-diodes, transforming them into electrical signals to be processed in a DSP.

### Piezoresistive MEMS sensors

Advances in manufacturing procedures, biocompatible materials and encapsulation have qualified MEMS sensors to be used in biomedical applications [62–65]. MEMS sensors are made using materials and micro-machining techniques originated in the microelectronics industry, and are based primarily on silicon technology [66, 67].

The first prototype of an implantable MEMS sensor for TIHAs was the piezoresistive MEMS accelerometer (D) to be implanted on the incus developed by Park et al. in 2007 [16].

The accelerometer proposed by Park et al. used piezoresistive transduction because of its low output impedance, enabling remote amplification circuitry. A prototype (387 × 800 × 230 μm, $m=166\,\upmu$g) was fabricated with a silicon proof mass suspended by a thin flexible beam and piezoresistors coupled on each lateral face of the beam, so that the acceleration induces differential strain by the shear stress induced on the elements. The sensor development also included the design of flexible electrical wiring in order to minimally affect the stiffness of the system, and a packaging solution which increased the dimensions of the sensor in only a couple of micrometers. The prototype was tested in a laboratory set-up to measure the influence of packaging techniques on damping properties. It was also tested using human cadaveric TBs, comparing the sensor response with the results from a laser Doppler vibrometer (LDV) measurements of the velocity of the incus (where the accelerometer was mounted) and the stapes.

### Capacitive sensors

Another mechanism used for sensors implanted in the ME is capacitive transduction, used in many applications [68, 69] including vibro-acoustical sensors. These include the microphone approach described previously, but also more specific sensors, like acoustical directivity sensors [70] or vibration sensors that are sensitive to displacement, velocity or acceleration.

**Fig. 5:**
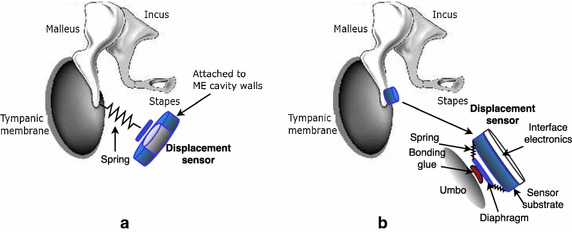
Capacitive MEMS displacement sensor schemes of **a** the capacitive MEMS displacement sensor (E1) connected to the umbo by a spring, and **b** the capacitive MEMS displacement sensor (E2) coupled directly to the umbo (Based on information from [20, 22])

A capacitive MEMS displacement sensor (E1), was proposed by Huang et al. in 2007 [22] as CI and TIHA sensor. The prototype shown in Fig. 5 employs a coiled spring (*m* = 15 mg, *k* = 10 N/m) to transmit the umbo displacement to the membrane of an ECM (WM-65A103), which is attached to the ME cavity wall. This way the microphone acts as a displacement sensor. The study was focused on the spring design in order to maximize the signal amplitude obtained, and the sensor was tested in a laboratory model using a lead zirconate titanate (PZT) shaker and a laser doppler vibrometer (LDV) to measure the velocity of the umbo. The prototype was also tested in a single human TB.

In a later study (2009), Ko et al. [20] compared the sensor (E1) with another capacitive MEMS displacement sensor (E2), coupled directly to the umbo (Fig. 5b) to solve design and mounting problems present with sensor E1. The E2 prototype consists of a silicon diaphragm hold in place with a set of springs of negligible mass. The other ends of the springs are connected to a silicon substrate with a mass of 20 mg, which vibrates along with the umbo. Capacitance changes between the diaphragm and the substrate are converted into electrical signals trough an amplifier circuit. The prototype was fabricated on a $2\times 2\,\text {mm}$ silicon chip (total $m=25\,\text {mg}$) also containing the interface circuit, and was tested in human TBs.

**Fig. 6:**
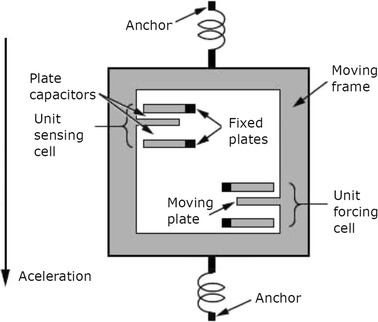
Capacitive MEMS accelerometer typical operating scheme of a capacitive MEMS accelerometer

A capacitive MEMS accelerometer (F1) attached to the umbo was proposed in 2007 by Zurcher et al. [21]. Capacitive MEMS accelerometers are widely used in industry due to its greater dynamic range and low SNR when compared to piezoresistive or piezoelectric devices  [68, 71] and Fig. 6 shows a typical operating scheme of a capacitive MEMS accelerometer. The movable plate (along with the proof mass) generates a capacitance change between the fixed plates, which can be measured with differential capacitance-to-voltage circuitry [21].

Zurcher’s prototype was fabricated with silicon on insulator technology (SOI), laid on an area of $1\times 1\,\text {mm}^2$ with a prof mass of 14 mg. Including the interface electronics it has a total size of $2.5 \times 6.2\,\text {mm}$, and a packaged mass of about $m = 25\,\text {mg}$. The accelerometer was attached to the umbo of a human TB, with the incus removed. Simultaneously acceleration of the umbo was measured with a LDV to analyze accuracy and effects of mass loading. Sensor performance was also compared with the performance of MEMS displacement sensors E1 and E2 [20], previously introduced.

In a further work (2012), Young et al. [40] analyzed the encapsulating conditions of the capacitive MEMS accelerometer F1, measuring its response under vacuum conditions. The study pointed to the need of reducing packaging size, analyzing encapsulating conditions and bio-compatibility of materials.

A more recent work on a capacitive MEMS accelerometer (F2) was published by Sachse et al. in 2013 [23]. A lumped parameter model of the sensor was developed in order to consider mechanical and electrical noise, and to calculate the resonance frequency. A test sample fabricated with SOI technology with an active area of $2.1\times 2.1\,\text {mm}^2$ was tested in human TBs in order to validate the model and analyze its response.

A capacitive microphone (G), to be implemented in the ME and to measure the pressure variation inside the ME cavity caused by the tympanic membrane vibration, was proposed by Woo et al. in 2012 [53]. Diameter (10 mm) and thickness ($20\,\upmu$m) of the stainless steel SUS316 membrane were defined using an electrical circuit model. A FE model of the microphone was developed to obtain its response to the sound pressure in the ME cavity. A calibrated ECM (OB-3111, BSE co.) was implanted in the ME of guinea pigs [12], in order to compare the performance and implantation feasibility of such a microphone with other implantable sensors, obtaining a smaller transmission loss if compared to the subcutaneous microphone [49].

Recently, MEMS technology has also been used to develop capacitive sensors to measure the intra-cochlear pressure [72]. It has been suggested that such designs could be later considered as an option for implantable sensors for hearing devices. However, such alternative has not been evaluated in the literature and will not be further discussed here.

### Piezoelectric sensors

Piezoelectric materials generate electrical voltage when deformed (direct piezoelectric effect) and show mechanical deformation when an electrical voltage is applied (inverse piezoelectric effect), which enables the piezoelectric transducer to act as sensor or actuator, respectively. The piezoelectric principle can be used in force transducers, accelerometers and microphones, and have also been proposed for implantable sensors.

Piezoelectric force transducers usually employ a cantilever bimorph (two layers of piezoelectric ceramic on either side of a stiffening material). The first prototype of a piezoelectric force sensor (H1) to be used as TIHA sensor was proposed by Javel et al. in 2003  [14]. In the study, a prototype was constructed from raw piezoelectric bimorph material cut into rectangular cantilever shapes ($7\times 1\,\text {mm}^2$). The sensor was implanted on the malleus of adult cats, and its response was compared with the vibration measured with a LDV with its laser pointed to the tip of the beam.

**Fig. 7:**
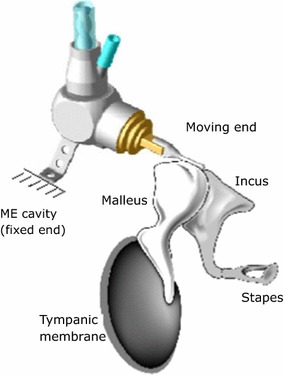
Esteem scheme of *Esteem* sensor (Adapted from Envoy Medical)

A piezoelectric force sensor is also being used in *Envoy Medical*’s *Esteem* device (H2). The device was approved by the FDA in 2011 and until 2014 a total of 134 implants are reported in the literature [25]. It is actually the only commercial TIHA to that relies on the measurement of the ossicles’ vibration as a representation of the external sound field. In *Envoy Medical*’s solution the piezoelectric force sensor is deformed by the incus vibration, transforming this deformation into electrical voltage to be processed in a DSP and sent to a piezoelectric actuator fixed to the stapes. While technical characteristics of the sensor are not detailed in the literature, it is clear that it acts as a force transducer, similar to sensor H1, being fixed to the wall of the ME cavity (with glass ionomer cement) and having its moving part attached to the incus [54], as shown in Fig. 7. A small amplifier circuit is located on the basis of the sensor to transform the high impedance of the transducer to the lower impedance required for signal acquisition.

**Fig. 8:**
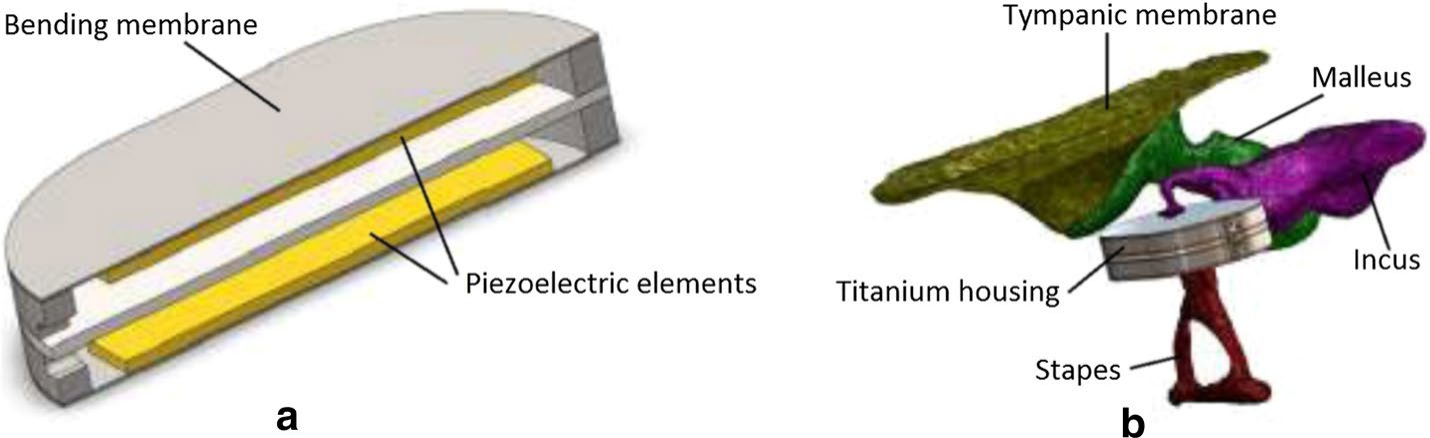
Piezoelectric force sensor ** a** Cross-sectional view of the piezoelectric force transducer (H3). **b** FE model of the force transducer (H3) coupled to the ME (Adapted from [24], under the Creative Commons Attribution License)

Another type of a piezoelectric force transducer (H3) was presented by Koch et al. in 2013 [55]. The study proposed a bidirectional membrane transducer, to be inserted at the incudostapedial joint (Fig  8b) and to sense the force transmitted through this joint. The prototype was assembled from two identical titanium housings capped with two membranes mounted in a single unit (Fig.  8a). A piezoelectric element is glued to the inside of the membranes, the latter acting as bending plates. One of them is used as a sensor, the other one as an actuator. The total size of the oval-shaped transducer is $4\times 2.5\times 1\,\text {mm}^3$, and its total mass is $m= 35\,\text {mg}$. A FE model of the device was developed, and a prototype was tested in TBs, having its response measured with a low-noise preamplifier (SR560), and in a set-up simulating the human ME with synthetic materials [24].

**Fig. 9:**
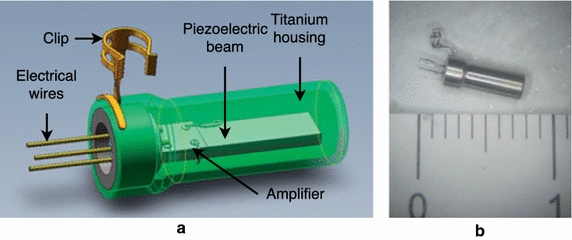
Piezoelectric accelerometer **a** scheme of the accelerometer (I) and its parts. **b** Photograph of the prototype (I) (Adapted from [73] with permission of Taylor & Francis Ltd, http://www.tandfonline.com on behalf of Acta Oto-Laryngologica AB (Ltd))

A biocompatible piezoelectric accelerometer (I) was proposed by Kang et al. in 2012 [13]. The prototype consists of a piezoelectric ceramic bimorph element and a chip containing a preamplifier (LMV 1032), both encapsulated in a titanium box, having a total size of $4.5 \times 1 \times 0.3\,\text {mm}^3$ and a total mass of $38.4\,\text {mg}$. The response of the prototype glued to the incus of cats when applying an acoustical stimuli was measured. The study also suggests the possibility of using MEMS technology in order to reduce the sensor size. Later in 2013, Ga et al. [56] modeled the same sensor using the FE method, simulating its response when implanted in the human middle ear. More recently in 2016, Jia et al. [73] placed the piezoelectric accelerometer I into a thin titanium tube with a clip, in order to seal the front part of the sensor (Fig. 9), and to be coupled to the long process of the incus. This modification increase its size to $5.91 \times 2.4 \times 2.02\,\text {mm}^3$, and its mass to $67.0\,\text {mg}$. The new prototype was tested in seven cadaveric temporal bones.

Piezoelectric MEMS accelerometers as sensors for implantable hearing devices have been reported in the literature due to its potential to reduce the sensor size. Such sensors have employed various materials, such as PZT, AlN, ZnO, or PVDF, and show a bandwidth of up to 20 kHz and sensitivities comparable to capacitive MEMS accelerometers [71]. The most typical configuration for piezoelectric MEMS accelerometers is an inertial mass attached to a cantilever beam. The deformation of a piezoelectric layer at the beam base generates an electrical voltage or charge potential. Inertial mass and beam are made of silicon and a thin layer of piezoelectric material, deposited on the beam. The dimensions of the inertial mass and beams are chosen by design to provide the desired dynamic range and sensitivity.

The first piezoelectric MEMS accelerometer (J1) to be used as CI sensor was proposed by Beker et al. in 2013 [19]. The study presented a FE model, validated experimentally through a prototype using a silicon base and a PZT layer. The prototype’s total size is $4.25\times 4\times 0.525\,\text {mm}^3$. The study also tentatively suggests that the sensor may harvest the energy generated by the umbo movement, in order to be used in other CI stages.

More recently, Yip et al. [18] also presented a piezoelectric MEMS accelerometer (J2) to be used as a CI sensor. In his work the voltage output of a prototype manufactured with piezoelectric PZT-5A ceramic was measured in a laboratory set-up. This voltage was used later in an electrical model which included an amplification circuit. Another prototype was made by the same authors using sensor-on-chip (SoC) technology, having its charge amplification circuit integrated on the chip. This last prototype was coupled to the umbo in human temporal bones, and features a signal processing algorithm in order to reduce CI power consumption. The technical characteristics of the sensor were not detailed by the authors, which focused its description on the characteristics of the amplification circuit required to process the high impedance at the sensor output.

## Sensor performance comparison

The performance of the implantable sensors presented in the previous section is now compared from two perspectives: (1) technically in terms of sensitivity, frequency response, bandwidth, EIN, SNR and energy consumption and (2) regarding implantation and operation problems, such as experiences in tests or patients’ satisfaction.

### Technical performance characteristics

The most important technical performance characteristics of the implantable sensors reviewed in this article are presented in Table 2. Although most of the information was taken from the literature related directly to the sensors, some data had to be estimated and are further commented in the table notes. Sensitivity and SNR (in dB ref. 1 mV/Pa) are normalized to an input RMS sound pressure of 1 Pa (94 dB SPL) at 1 kHz.

**Table 2:** Principal technical performance characteristics of implantable sensors for hearing devices

Sensor type	ID	Bandwidth (kHz)	Sensitivity (dB ref. 1 mV/Pa)	SNR (dB ref. 1 mV/Pa)	EIN (dB SPL)	Power consumption (mW)
Subcutaneous mic.	A1	0.1–10	5	–	–	0.05–0.5^a^
Subcutaneous mic.	A2	0.2–6	− 10	–	–	0.05–0.5^a^
Subcutaneous mic.	A3	0.25–5	–	–	–	0.05–0.5^a^
Subcutaneous mic.	A4	0.1–8	35	–	–	0.05–0.5^a^
Electromagnetic sensor	B	0.25–3	− 30	37	57	$\approx$ 1
Optical sensor	C	0.5–10	− 46^b^	–	–	6.4
Piezoresistive MEMS accelerometer	D	0.9–7	6^c^	40	63	> 1
Capacitive MEMS displacement sensor	E1	0.5–5	20	55	60	$\approx$ 4.5
Capacitive MEMS displacement sensor	E2	0.8–8	30	70	34	$\approx$ 4.5
Capacitive MEMS accelerometer	F1	0.2–6	19	35	35	$\approx$ 4.5
Capacitive MEMS accelerometer	F2	0.5–6	− 9^d^	70	24	–
Capacitive microphone	G	0.1–10.0	28	18	29	$\approx$ 1
Piezoelectric force transducer	H1	0.5–10	45	–	–	–
Piezoelectric force transducer	H2	0.25–8	–	–	–	–
Piezoelectric force transducer	H3	0.4–4	− 15	60	20	–
Piezoelectric accelerometer	I	0.25–10	1	52	38	0.12^e^
Piezoelectric MEMS accelerometer	J1	0.5–2.5	62^b^	–	–	–
Piezoelectric MEMS accelerometer	J2	0.3–6	20	50	44	0.01

^a^Power consumption of ECMs for hearing aids (diaphragm diameter 2.5–10 mm) [6, 46]

^b^Calculated with 0.34 mm/s/Pa, from the umbo velocity transfer function at 1 kHz  [74]

^c^Sensitivity of 0.02 V/$g_0$ obtained from a similar piezoresistive sensor  [17]

^d^Typical capacitive sensitivity is $1\times 10^7\,\text {mV/m}$ [75]

^e^Data for preamplifier LMV1032, $60\,\upmu \text {A}$ current, 2 V voltage supply [76]

**Fig. 10:**
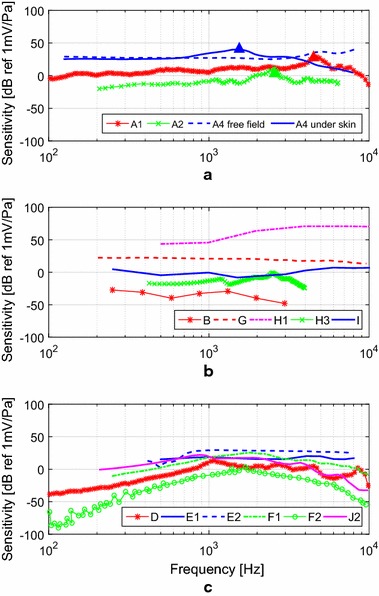
Frequency responses sensitivity frequency responses of **a** the subcutaneous microphones A1 [9] and A2 [10] under patients’ skin, microphone A4 in a free field and under the skin [50]; **b** the electromagnetic sensor B [51] in human TBs (malleus), the capacitive microphone G in guinea pigs (ME cavity) [12], the piezoelectric force transducers H1 in cats (incus) [14] and H3 in a synthetic ossicular chain (incudostapedial joint) [55], the piezoelectric accelerometer I in TBs (incus) [73]; **c** the piezoresistive MEMS accelerometer D (incus) [16], the capacitive MEMS displacement sensors E1 [22] and E2 [20] in human TBS (umbo), the capacitive MEMS accelerometers F1 [21] and F2 [23] in human TBs (umbo), and the piezoelectric MEMS accelerometer J2 [18] in human TBs (umbo)

The sensitivity frequency responses presented in the literature, mainly those obtained from implanted sensors (in patients, in human TBs or in animals) are shown in Fig. 10. They are separated in order to facilitate their visualization and to be compared according to similar mechanisms: Fig. 10a for subcutaneous microphones, Fig. 10b for sensors implanted in the ME and Fig. 10c for MEMS sensors.

**Fig. 11:**
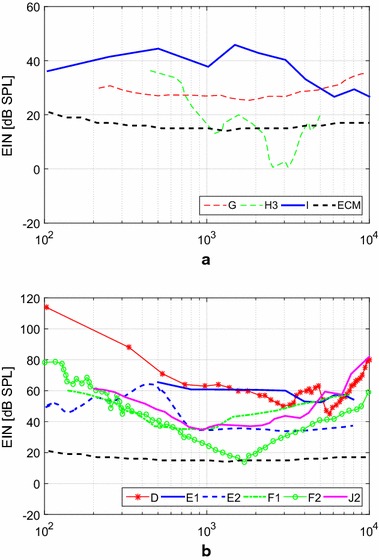
Equivalent input noise (EIN) equivalent input noise of **a** the capacitive microphone G in guinea pigs (ME cavity) [12], the piezoelectric force transducers H3 in a synthetic ossicular chain (incudostapedial joint) [55], the piezoelectric accelerometer I in TBs (incus) [73]; **b** the piezoresistive MEMS accelerometer D (incus) [16], the capacitive MEMS displacement sensors E1 [22] and E2 [20] in human TBS (umbo), the capacitive MEMS accelerometers F1 [21] and F2 [23] in human TBs (umbo), and the piezoelectric MEMS accelerometer J2 [18] in human TBs (umbo). Both figures include the EIN of ECM used in conventional HAs [7]

Figure 11 shows the frequency behavior of the EIN of the implantable sensors, considering a typical cochlear implant frequency discretization of 100 samples per second. The EIN analysis is well suited approach to investigate sensor performance, since it determines the minimum detectable SPL in a given frequency range, whereas a sensitivity analysis does not consider the sensor’s internal noise. The EIN of an ECM used in conventional HAs [7] is included as reference for performance comparison. For sensors D, E1, J2 and I, the EIN was estimated from available spectral noise data, whereas for the other sensors, such data can be taken directly from the literature. In the case of the subcutaneous microphones (A1, A2, A3 and A4), no results for EIN are provided, since most of the literature on these sensors focuses on the implanted device, therefore including the effects of the processor, DSP, actuator and even the patient. For sensors B and G, there was insufficient information to estimate its EIN frequency behavior.

Performance characteristics of the subcutaneous microphones given in Table 2 vary mainly due to different measurement conditions and microphone characteristics (diaphragm area and stiffness). While all microphones meet the requirement related to the minimum frequency range (from 200 Hz to 5 kHz), *TICA*’s microphone (A1) has a considerably larger bandwidth of approximately 10 kHz. The subcutaneous microphone A4 has the greatest sensitivity (35 dB), which is probably due to the larger diameter of the diaphragm (12 mm), when compared to the *TIKI* microphone A2 ($<5\,\text {mm}$) and the *TICA* microphone A1 ($<5\,\text {mm}$). Nevertheless, this sensitivity comparison is biased due to the unknown characteristics of their amplification circuits, which largely influence these responses [41]. The frequency response of *Carina*’s subcutaneous microphone (A3) was not found in the literature.

From Fig. 10a it can bee seen that all sensitivity frequency responses measured for the implanted subcutaneous microphones (A1, A2, A4) show a light increase in sensitivity up to the resonance frequency and a roll-off for larger frequencies. This roll-off, caused by the effect of skin over the microphone, was analyzed by Jung et al. [50], comparing the sensitivity response of the subcutaneous microphone A4 under free field conditions and implanted under artificial skin. In the case of sensor A4, the skin load shifts the resonance frequency from the original 5 kHz to approximately 1.5 kHz, causing a difference in the sensitivity behavior above 600 Hz.

The sensitivity roll-off was observed for frequencies larger than 2600 Hz in the *TIKI*’s subcutaneous microphone A2 [10], whereas the same happens for frequencies larger than 4.5 kHz in the case of *TICA*’s subcutaneous microphone A1 [9].

While information regarding the sensitivity response is often available for subcutaneous microphones, sensor noise, EIN and power consumption of such sensors is not detailed in the literature. Power consumption between 0.05 and 0.5 mW [6, 46], can be considered as a reference, and would be acceptable for implantable sensors. Nevertheless, acceptable power consumption of the sensor also depends on the entire hearing device’s power consumption.

For sensors implanted in the middle ear, performance characteristics vary according to their transduction mechanism and configuration. Sensitivity frequency responses of sensors implanted in the middle ear (except for MEMS sensors) are shown in Fig. 10b), and these sensors’ EIN curves are shown in Fig. 11a.

The electromagnetic sensor B tested in TBs exhibits low sensitivity, e.g. at 3 KHz sensitivity is as low as − 30 dB. According to Maniglia et al. [51], a reduction of bandwidth after implantation is explained by the load effect of the magnet in the ossicular chain, which reduces its natural frequency. Although the EIN frequency behavior could not be obtained, SNR and EIN at 1 kHz, and power consumption were given in the original paper [51], as shown in Table 2. A 0.25 to 3 kHz bandwidth was achieved for SPLs in excess of 57 dB and power consumption was over 1 mW, not meeting the minimum requirements presented previously.

The optical sensor prototype, C, was tested with a piezoelectric shaker in a laboratory set-up at a single frequency of 5 kHz, although a wide bandwidth has been specified. The authors claim that a bandwidth from 500 Hz to 10 kHz was achieved, but the paper lacks the information to confirm these claim. Assuming that this sensor would be coupled to the umbo, where a velocity of 0.34 mm/s$^2$ at 1 kHz is achieved for 94 dB SPL applied to the tympanic membrane [74], the estimated sensitivity is − 46 dB, by far the lowest for all implantable sensors reviewed in the current article (Table 2). While information about sensor noise is not available, an important disadvantage of the optical sensor C is its high power consumption of $\approx \,6.4\,\text {mW}$ with a laser supply current of 80 mA [52], which is more than ten times the acceptable maximum (0.5 mW).

The capacitive microphone G implanted in the ME cavity has a very flat sensitivity frequency response, featuring 28 ± 3 dB in a wide bandwidth from 100 Hz to 7 kHz (Fig. 10b). Thus, its sensitivity outperforms most of the capacitive sensors implanted in the middle ear. Regarding the sensor noise (Fig. 11a), the capacitive microphone G has a low and flat EIN curve (29 ± 4 dB) in a large bandwidth from 100 Hz to 10 kHz. Therefore, this sensor achieves the specified requirements for implantable sensors regarding bandwidth and EIN. The only drawback of this sensor, regarding the requirements, is its power consumption, estimated to be $1\,\text {mW}$, being slightly higher than the maximum 0.5 mW estimated from traditional ECM.

As shown in Table 2, the piezoelectric force transducer H1 implanted on the cat’s incus, has a considerably higher sensitivity (45 dB at 1 kHz) when compared to the capacitive sensors implanted in the ME, although the capacitive microphones present a flatter sensitivity response.

Information on sensor’s H1  [14] noise characteristics and power consumption is not available, although the latter is usually lower than 0.5 mW due to its piezoelectric operation, which consumes less power in the amplifying circuit [67, 77].

For the second piezoelectric transducer, *Esteem*’s force transducer (H2), only very few technical details can be obtained from the literature, which focuses on clinical results [25] and no frequency sensitivity or EIN curve can be plotted. The only non-clinical information about this sensor is related to the bandwidth, spreading from 250 Hz to 8 kHz [15].

The third piezoelectric force transducer (H3) exhibits a sensitivity curve, measured in the laboratory, that is relatively flat until 3 kHz [24]. Since Koch did not develop a amplifying circuit, sensitivity is mere − 15 dB at 1 kHz. EIN drops from 35 dB at 500 Hz to 20 dB at 1 kHz, as shown in Fig. 11a, but an amplifier circuit might induce more noise into this sensor, and therefore increase EIN. As sensor H3 operates alongside an actuator in the same device, there is a strong feedback during operation, limiting its upper frequency to 4 kHz. In addition, feedback control may require more operating power.

A low sensitivity of 1 ± 5 dB and flat response curve was obtained for the piezoelectric accelerometer I, which was implanted on the incus of seven TBs [73]. Its EIN varies between 28 dB and 45 dB SPL in the 100 Hz to 10 kHz frequency band, which is slightly higher than the specified 30 dB maximum EIN SPL. While the piezoelectric accelerometer I has a low power consumption of 0.12 mW, its dimensions of $5.91 \times 2.4 \times 2.02\,\text {mm}^3$, and its mass of $67.0\,\text {mg}$, limit its applicability in the middle ear cavity.

The sensitivity response functions of MEMS sensors implanted in the ME are shown in Fig. 10c and their EIN curves are shown in Fig. 11b. The piezoresistive MEMS accelerometer D shows an almost flat response in a band from 900 Hz to 7 kHz, and its sensitivity of 6 dB at 1 kHz is one of the lowest reported for implantable MEMS sensors. Also, piezoresistive sensors tend to exhibit high noise and high power consumption [23], well exemplified in Park’s sensor with a high EIN of 63 dB at 1 kHz and power consumption estimated as $1\,\text {mW}$. Therefore, although Park developed a small and light sensor ($387 \times 800 \times 230\,\upmu$m$^3$, $m= 166\upmu$g) with flexible wires and an efficient packaging solution, its inherently high noise and power consumption limits this technology’s feasibility.

The capacitive MEMS displacement sensor E1 tested in TBs  [22] has a flat sensitivity response (20 ± 2 dB) in a limited 500 Hz to 5 kHz bandwidth. Its EIN curve is approximately flat varying from 64 dB at 500 Hz to 56 dB at 8 kHz, values in excess of approximately 30 dB of the limit established previously.

Ko et al. further improved the capacitive MEMS displacement sensor E1 through the second generation device E2 [20], featuring a higher sensitivity (30 dB at 1 kHz) and, most importantly, a lower EIN of 34 dB SPL from 800 Hz to 8 kHz (Fig. 11b), although the EIN curve shows a distinctive 66 dB SPL peak at 450 Hz. Both E1 and E2 consume similarly 4.5 mW.

The capacitive MEMS accelerometer F1, evaluated in TBs, exhibits a highly frequency dependent sensitivity behavior, varying from − 15 dB at 250 Hz, to 19 dB at 1 kHz and 3 dB at 8 kHz, as seen in Fig. 10c. Since the noise’s spectral density is inversely proportional to the sensor sensitivity [21], among other parameters, a strongly varying sensitivity causes a large variation in the EIN, as seen in Fig. 11b, provided the others parameters are not altered in the same order of magnitude [23]. For instance, EIN at 250 Hz is 54 dB, at 1 kHz EIN is 35 dB and at 8 kHz it reaches 58 dB SPL again.

The capacitive MEMS accelerometer F2 [23] was designed to operate with a natural frequency of 1.7 kHz, which generated a dip in the EIN curve for this frequency. Due to the large variation in sensitivity (− 50 dB at 250 Hz, 2 dB at 1.7 kHz and − 45 dB at 8 kHz) as shown in Fig. 10c, this sensor’s EIN declines steadily from 45 dB at 250 Hz to 16 dB at 1.7 kHz before rising again up to 44 dB at 8 kHz. Therefore, sensor F2 is able to detect 30 dB SPL only between 750 Hz and 2.8 kHz, and SPLs above 40 dB SPL between 500 Hz and 6 kHz, as shown in Table 2. Power consumption, however, was not reported and there is no sufficient information for a proper estimate.

The piezoelectric MEMS sensor J1 was tested using solely a shaker [19], and no further analysis was made. The sensor is mainly characterized by a high sensitivity (62 dB, estimated from the umbo vibration) in a limited bandwidth. In a laboratory set-up, the sensor was capable to generate $1.3\, \upmu \text {W}$ of power, for an acceleration of $9.8\,\text {m/s}^2$ at 474 Hz. Nevertheless, since sensor J1 was not tested in TBs and its power consumption and SNR were not measured or specified, the performance can hardly be analyzed and compared to the other sensors.

For the piezoelectric MEMS accelerometer J2 sensitivity was measured to be 20 dB when the sensor was coupled to the umbo in TBs [18]. Sensor J2 features a 700 Hz to 2.2 kHz bandwidth and EIN equals 40 dB at 1 kHz, as shown in Fig. 11b. For SPLs larger than 50 dB, the sensor is capable of detecting sounds in the 350 Hz to 4.5 kHz frequency band. Voltage output was measured after addition of a charge amplification circuit ($10\,\upmu \text {W}$), and the total energy consumption of the sensor with the amplification circuit was $572\,\upmu \text {W}$, which is within the limits established for the energy consumption.

### Performance effectiveness and limitations

When a sensor is implanted its overall, and thus effective, performance is defined by both the technical performance characteristics, reviewed in the previous section, and a variety of other aspects such as long term effectiveness and surgical issues, many of them interrelated. Most aspects are related, directly or indirectly, to the lieu of implantation and the transduction principle used. In the following section some of these aspects will be analyzed.

Subcutaneous sensors and transducers for the middle ear incur on different challenges regarding surgical methods and complications. For example, *TICA*’s subcutaneous microphone (A1) was implanted under the skin of the external ear canal in order to take advantage of the directional filtering characteristics of the pinna, and its construction contributes to the suppression of body noise [9]. However, interference due to sound reflection at the tympanic membrane is a major issue in *TICA*’s sensor, that will eventually pick up the reflected wave [2, 9].

Another problem was sensor degradation and scar formation in the skin of the ear canal, which tended to affect the sensitivity of the subcutaneous microphone at higher frequencies that could not be correctly predicted.^3^

In the case of the *TIKI* device, that uses sensor A2, variation of skin stiffness with time after implantation surgery influenced the performance of the subcutaneous microphone, resulting in a decrease of the hearing threshold after 6 months. The benefit reported in the three patients which used the *TIKI* device was lower than that reported when the CI uses an external microphone.

The most relevant problems related to *Carina*’s subcutaneous microphone (A3) are: perception of body noise [48], skin infections and partial extrusion of the device [25]. Furthermore, feedback influenced *Carina*’s functional gain, limiting its bandwidth to 4 kHz, affecting therefore the selection criteria [26]. It should be noted, however, that these problems could not be attributed only to the sensor used in *Carina*, since the actuator and DSP also modify the device’s performance.

Sensors implanted in the middle ear usually capture the vibrations produced by the external sound after conversion into vibration by the tympanic membrane. This approach avoids the interference of body noises and preserves the directional filtering characteristics of the external ear [31]. As shown in the previous section, sensors implanted in the middle ear include a large variety of transducer principles, along with a microphone configuration (sensor G). Implanting the microphone in the middle ear cavity could be an alternative solution to the problem of increased attenuation caused by the skin over the microphone. In general, sensors implanted in the middle ear avoid the interference of body noises and preserve the directional filtering characteristics of the external ear [31], however other surgical difficulties arise from such choice. One of the problems reported for all sensors implanted in the middle ear is the complexity of implantation, which is directly related to the size of the components and the available space, which is usually assumed to be a maximum 2 mm edge cube, for a sensor with an accelerometer configuration [23].

In the case of the electromagnetic sensor B, close proximity between the magnet and the coil limits the choice of the lieu of implantation in the middle ear cavity, and the electromagnetic sensor impedes the patient to do MRI scans. The solution using the optical sensor C and the Microphone G, suffer from fiber holder instability due to body movements, which could affect the accuracy and precision of the response measured in the middle ear [12, 52].

Difficulties in the implantation procedure were also reported for the transducers E1, H1, H2 and H3, since they all need to be mounted close to the middle ear cavity wall, having its spring aligned in such a way as to provide the proper spring compression.

In the case of *Esteem*’s sensor (H2), the literature also reports implantation problems, such as necessity of repositioning and removal of the sensor [15]. Further on, facial paralysis and partial loss of taste after surgery have been reported, due to the need to open a large entry in order to fit the large sensor and actuator in the ME cavity, which incurred a small facial nerve damage [54].

The influence of the sensor on the middle ear dynamics and complexity of its implantation could be reduced with the accelerometer configuration. In order to be implanted in the ossicular chain, the sensor should have a maximum dimension of 2 mm, certainly achievable for MEMS accelerometers and displacement sensors. However, up to the moment, none of these MEMS sensors was able to ally size reduction with all technical performance criteria, as is further detailed in the conclusions.

Also, the use of MEMS accelerometers and MEMS displacement sensors will incur new surgical and long term issues. For instance, for both sensors, flexible wire connectors will have to be developed in order to minimize the influence on the ossicular chain’s dynamic response, which would also affect the sensor’s response. Additionally, wire connections and its properties should remain unaffected over time, in order to avoid variation in sensitivity over time. Furthermore, the packaging and fixation method has to be developed in parallel, since it must avoid issues such as rejection and guarantee bio-compatibility, hermeticity and non-toxicity.

## Conclusions

Different technological approaches for implantable sensors for totally implantable hearing devices are found in the commercial TIHAs *Carina* and *Esteem*, and in various research prototypes. However, information about sensor performance and project requirements are scattered or incomplete in the literature.

Therefore, in this technical review, the first aspect approached was the investigation of the general requirements for implantable sensors, carried out in sight of the two main categories of implantable transducers: subcutaneous microphones and sensors installed in the middle ear. This analysis rendered for the subcutaneous microphone the following recommendations: bandwidth from 100 Hz to 8 kHz, dynamic range from 30 dB to 100 dB SPL and a power consumption lower than 0.5 mW. For sensors implanted in the middle ear the same bandwidth and power consumption is recommended, although dynamic range should be from 40 dB to 100 dB SPL and maximum dimensions should be considered, alongside other recommendations for packaging and wiring.

A general classification for implantable sensors was proposed in Fig. 2, and a brief description of each sensor’s development stage was summarized in Table 1. All selected implantable sensors were then throughly presented with their most important geometries characteristics.

Regarding subcutaneous microphone performance, it was seen that the microphones’ sensitivity increases with the diaphragm size , although it also depends on other characteristics. In this case, the EIN analysis become more difficult since there is no data available in the literature for these microphones, except when fitted into hearing aids.

It was seen that the use of subcutaneous microphones presents a series of limitations, such as: (i) body noise interference; (ii) sensitivity variation over time due to scar tissues; (iii) lack of directionality, or larger power consumption in trying to account for directionality; and (iv) feedback noise (reported in some cases).

Middle ear implantable sensors are analyzed mainly through Fig. 11, where EIN is shown over frequency for all these sensors. In Fig. 11a EIN is shown for the non-MEMS sensors that are implanted into the middle ear. Among these, the capacitive microphone G implanted in the middle ear cavity exhibits a low and flat EIN response curve (29 ± 4 dB) in a large bandwidth from 100 Hz to 10 kHz. Therefore, this sensor achieves the specified requirements for implantable sensors regarding bandwidth and EIN, although its rather large 1 mW power consumption.

The piezoelectric force transducer H3 exhibits a prominent result regarding EIN, yet allying low power consumption, which can explain that a similar sensor is being used in the commercial device Esteem. However, surgical difficulty is high with this sensor and device, as two support points in the middle ear are required, and alignment should be very accurate. Some of the problems reported in the literature for the Esteem are associated with these aspects, such as the need for repositioning and removal of the sensor [15], temporary facial paralysis and partial loss of taste after surgery, the latter being due to the necessity of opening a large insertion in the temporal bone in order to fit this larger sensor. The other non-MEMS piezoelectric sensor, microphone I, also exhibits a promising result regarding piezoelectric transduction, being able to measure 40 ± 8 dB SPL between 200 Hz and 5 kHz consuming little power, although its larger dimensions and previously related problems associated to implantable microphones must be considered.

In Fig. 11b it is noticeable that the piezoresistive sensor (D) performs with the highest EIN over the entire frequency range. This behavior is due to the intrinsically high noise of the piezoresistive effect. It is worth noting, however, that this sensor is much smaller ($387\times 800\times 230\, \upmu$m), therefore, a fairer comparison would be between sensors of the same size.

Regarding the capacitive displacement sensors E1 and E2, it is possible to see that the second generation sensor E2 considerably improved performance over E1. While E1 was able to detect only 64 dB SPL at 500 Hz and 56 dB SPL at 8 kHz, sensor E2 was able to measure down to 34 dB SPL from 800 Hz to 8 kHz, although a rise in EIN to 66 dB SPL at 450 Hz compromises sensor E2 at low frequencies. The capacitive MEMS accelerometer F1 and the piezoelectric MEMS accelerometer J2 perform similarly to the sensor E2. Regarding only MEMS sensors, these three designs (E1, E2, F1) are surpassed only by the capacitive MEMS accelerometer F2, which was able to measure down to 45 dB at 250 Hz, 16 dB at 1.7 kHz and 44 dB at 8 kHz. Sachse achieved this improved behavior by forcing a lower natural frequency (1.7 kHz) which lowered the internal noise of the device in the frequency range and lowers EIN between 250 Hz and 8 kHz to values smaller than 45 dB. Nevertheless, it is crucial to notice that, by using the capacitive transduction mechanism, the devices E1, E2, F1 and F2 tend to consume up to 4.5 mW, whereas the piezoelectric accelerometer J2 may consume only 0.2 mW on the amplifying circuit (Table 2), thus limiting applicability over long periods of time.

### Closing remarks

The authors of this review paper see MEMS sensors coupled to the middle ear ossicular chain as the future for implantable sensors. To our knowledge, microphones are hardly going to be further improved, since it is an established technology to which very little could be changed for this application. MEMS sensors, however, are in an exciting new field, which should evolve over the next years to accommodate low noise sensors. MEMS piezoelectric and capacitive accelerometers and displacement sensors should be further developed as to obtain a lower than 30 dB SPL EIN between 100 Hz and 8 kHz.

## Footnotes

1 These speakers are often also called receivers.

2 Considering battery capacities ranging from 65 to 630 mAh with 1.4 V of voltage supply and average service of 200 h [47].

3 Despite receiving commercial approval in Europe, *TICA* lost financial support from Implex. After bankruptcy, the technology was purchased by Cochlear, with the intent of adapting the fully implantable technology to future cochlear implant designs [2].

## References

[1] Counter P (2008). Implantable hearing aids. Proc Inst Mech Eng Part H J Eng Med.

[2] Carlson ML, Pelosi S, Haynes DS (2014). Historical development of active middle ear implants. Otolaryngol Clin N Am.

[3] Tefili D, Barrault G, Ferreira AA, Cordioli JA, Lettnin DV (2013). Implantes cocleares: aspectos tecnológicos e papel socioeconômico. Revista Brasileira de Engenharia Biomédica.

[4] Cohen N (2007). The totally implantable cochlear implant. Ear Hear.

[5] Carlson ML, Driscoll CL, Gifford RH, McMenomey SO (2012). Cochlear implantation: current and future device options. Otolaryngol Clin N Am.

[6] Yamasaki T, Kodama H, Yasuno Y. Electret condenser microphones for hearing aids. In: 2008 13th international symposium on electrets. IEEE. 2008. p. 134.

[7] Conklin W. Leveraging microelectromechanical microphones inherent matching to reduce noise using multiple microphone elements. In: Proceeding if the international congress on acoustics ICA. 2013.

[8] Zenner HP, Leysieffer H, Maassen M, Lehner R, Lenarz T, Baumann J, Keiner S, Plinkert PK, McElveen JT (2000). Human studies of a piezoelectric transducer and a microphone for a totally implantable electronic hearing device. Otol Neurotol.

[9] Zenner HP, Leysieffer H (2001). Total implantation of the implex TICA hearing amplifier implant for high-frequency sensorineural hearing loss: the tübingen university experience. Otolaryngol Clin N Am.

[10] Briggs RJ, Eder HC, Seligman PM, Cowan RS, Plant KL, Dalton J, Money DK, Patrick JF (2008). Initial clinical experience with a totally implantable cochlear implant research device. Otol Neurotol.

[11] Jenkins HA, Atkins JS, Horlbeck D, Hoffer ME, Balough B, Arigo JV, Alexiades G, Garvis W (2007). US phase I preliminary results of use of the otologics MET fully-implantable ossicular stimulator. Otolaryngol Head Neck Surg.

[12] Woo S, Lee J, Park I, Song B (2013). Feasibility test of implantable microphone at middle ear cavity. Electron Lett.

[13] Kang H-Y, Na G, Chi F-L, Jin K, Pan T-Z, Gao Z (2012). Feasible pickup from intact ossicular chain with floating piezoelectric microphone. Biomed Eng online.

[14] Javel E, Grant IL, Kroll K (2003). In vivo characterization of piezoelectric transducers for implantable hearing aids. Otol neurotol.

[15] Chen DA, Backous DD, Arriaga MA, Garvin R, Kobylek D, Littman T, Walgren S, Lura D (2004). Phase 1 clinical trial results of the Envoy system: a totally implantable middle ear device for sensorineural hearing loss. Otolaryngol Head Neck Surg.

[16] Park W-T, O’Connor KN, Chen K-L, Mallon JR, Maetani T, Dalal P, Candler RN, Ayanoor-Vitikkate V, Roberson JB, Puria S (2007). Ultraminiature encapsulated accelerometers as a fully implantable sensor for implantable hearing aids. Biomed Microdevices.

[17] Park W-T, Partridge A, Candler RN, Ayanoor-Vitikkate V, Yama G, Lutz M, Kenny TW (2006). Encapsulated submillimeter piezoresistive accelerometers. J Microelectromech Syst.

[18] Yip M, Jin R, Nakajima HH, Stankovic KM, Chandrakasan AP (2015). A fully-implantable cochlear implant soc with piezoelectric middle-ear sensor and arbitrary waveform neural stimulation. IEEE J Solid-State Circuits.

[19] Beker L, Zorlu O, Goksu N, Kulah H. Stimulating auditory nerve with MEMS harvesters for fully implantable and self-powered cochlear implants. In: Solid-state sensors, actuators and microsystems (TRANSDUCERS & EUROSENSORS XXVII), 2013 transducers & eurosensors XXVII: the 17th international conference on. IEEE. 2013. p. 1663–6.

[20] Ko WH, Zhang R, Huang P, Guo J, Ye X, Young DJ, Megerian CA (2009). Studies of MEMS acoustic sensors as implantable microphones for totally implantable hearing-aid systems. IEEE Trans Biomed Circuits Syst.

[21] Zurcher M, Young D, Semaan M, Megerian C, Ko W. MEMS middle ear acoustic sensor for a fully implantable cochlear prosthesis. In: Micro electro mechanical systems, 2007. MEMS. IEEE 20th international conference on. IEEE. 2007. p. 11–4. 10.1109/IEMBS.2006.259239 17945982

[22] Huang P, Guo J, Megerian CA, Young DJ, Ko WH. A laboratory study on a capacitive displacement sensor as an implant microphone in totally implant cochlear hearing aid systems. In: Engineering in medicine and biology society, 2007. EMBS 2007. 29th Annual international conference of the IEEE. IEEE. p. 5691–4. 2007. 10.1109/IEMBS.2007.4353638 18003304

[23] Sachse M, Hortschitz W, Stifter M, Steiner H, Sauter T (2013). Design of an implantable seismic sensor placed on the ossicular chain. Med Eng Phys.

[24] Koch M, Eßinger TM, Bornitz M, Zahnert T (2014). Examination of a mechanical amplifier in the incudostapedial joint gap: FEM simulation and physical model. Sensors.

[25] Pulcherio JOB, Bittencourt AG, Burke PR, da Costa Monsanto R, de Brito R, Tsuji RK, Bento RF (2014). Carina® and Esteem®: a systematic review of fully implantable hearing devices. PloS ONE.

[26] Bittencourt AG, Burke PR, de Souza Jardim I, de Brito R, Tsuji RK, de Oliveira Fonseca AC (2014). Implantable and semi-implantable hearing aids: a review of history, indications, and surgery. Int Arch Otorhinolaryngol.

[27] Mitchell-Innes A, Morse R, Irving R, Begg P (2017). Implantable microphones as an alternative to external microphones for cochlear implants. Cochlear Implants Int.

[28] Heffner HE, Heffner RS (2007). Hearing ranges of laboratory animals. J Am Assoc Lab Anim Sci.

[29] Suzuki Y, Takeshima H (2004). Equal-loudness-level contours for pure tones. JASA.

[30] Wilson BS. Engineering design of cochlear implants. In: Cochlear implants: auditory prostheses and electric hearing. Springer; 2004. p. 14–52.

[31] Moller AR (2006). Hearing: anatomy, physiology, and disorders of the auditory system.

[32] Zeng F-G, Rebscher S, Harrison W, Sun X, Feng H (2008). Cochlear implants: system design, integration, and evaluation. IEEE Rev Biomed Eng.

[33] Sherlock LP, Formby C (2005). Estimates of loudness, loudness discomfort, and the auditory dynamic range: normative estimates, comparison of procedures, and test-retest reliability. J Am Acad Audiol.

[34] Zeng F-G, Grant G, Niparko J, Galvin J, Shannon R, Opie J, Segel P (2002). Speech dynamic range and its effect on cochlear implant performance. J Acoust Soc Am.

[35] Sessler GM, Hillenbrand J. Hearing aid microphones: from electret to piezoelectret transducers. 2011. p. 463–7

[36] Lewis J (2012). Understanding microphone sensitivity. Analog Dialogue.

[37] Valentino M. Microphone handbook. PCB piezotronics vibration division. 2005.

[38] Dillon H (2001). Hearing aids.

[39] Park WT, O’Connor KN, Mallon Jr JR, Maetani T, Candler RN, Ayanoor-Vitikkate V, Roberson JB, Puria S, Kenny TW. Sub-mm encapsulated accelerometers: a fully implantable sensor for cochlear implants. In: Solid-state sensors, actuators and microsystems, 2005. Digest of technical papers. TRANSDUCERS’05. The 13th international conference On, vol. 1, IEEE. 2005. p. 109–12.

[40] Young DJ, Zurcher MA, Semaan M, Megerian CA, Ko WH (2012). MEMS capacitive accelerometer-based middle ear microphone. IEEE Trans Biomed Eng.

[41] Littrell RJ. High performance piezoelectric MEMS microphones. Ph.D. thesis, The University of Michigan. 2010.

[42] Zhao F, Koike T, Wang J, Sienz H, Meredith R (2009). Finite element analysis of the middle ear transfer functions and related pathologies. Med Eng Phys.

[43] Chen H, Okumura T, Emura S, Shoumura S (2008). Scanning electron microscopic study of the human auditory ossicles. Ann Anat Anatomischer Anzeiger.

[44] Sim JH, Puria S (2008). Soft tissue morphometry of the malleus-incus complex from micro-ct imaging. J Assoc Res Otolaryngol.

[45] Cheng T. Mechanical properties of human middle ear tissues. Ph.D. thesis, University of Oklahoma. 2007.

[46] Lewis J (2013). MEMS microphone: the future for hearing aids. Analog Dialogue.

[47] Penteado SP, Bento RF (2013). Performance analysis of ten brands of batteries for hearing aids. Int Arch Otorhinolaryngol.

[48] Bruschini L, Forli F, Passetti S, Bruschini P, Berrettini S (2010). Fully implantable otologics MET Carina device for the treatment of sensorineural and mixed hearing loss: audio-otological results. Acta Oto-laryngol.

[49] Jung ES, Seong KW, Lim HG, Lee JH, Cho JH (2011). Implantable microphone with acoustic tube for fully implantable hearing devices. IEICE Electron Express.

[50] Jung ES, Shin DH, Seong KW, Lee JH, Lee JW, Cho HS, Kim MN, Cho JH. Measurement of directivity for the design of an implantable microphone implanted under an artificial skin model. In: Biomedical and health informatics (BHI), 2012 IEEE-EMBS international conference on. IEEE. 2012. p. 297–300.

[51] Maniglia AJ, Murray G, Arnold JE, Ko WH (2001). Bioelectronic microphone options for a totally implantable hearing device for partial and total hearing loss. Otolaryngol Clin N Am.

[52] Vujanic A, Pavelka R, Adamovic N, Kment C, Mitic S, Brenner W, Popovic G. Development of a totally implantable hearing aid. In: Microelectronics, 2002. MIEL 2002. 23rd international conference on, vol. 1. IEEE. 2002. p. 235–8

[53] Woo SA, Cho HS, Park IHL, Song BS. Implementation of implantable microphone in the middle ear cavity and telemetry module. In: Biomedical engineering international conference (BMEiCON). IEEE. 2012. p. 1–4.

[54] Kraus EM, Shohet JA, Catalano PJ (2011). Envoy Esteem totally implantable hearing system phase 2 trial, 1-year hearing results. Otolaryngol Head Neck Surg.

[55] Koch M, Seidler H, Hellmuth A, Bornitz M, Lasurashvili N, Zahnert T (2013). Influence of the middle ear anatomy on the performance of a membrane sensor in the incudostapedial joint gap. Hear Res.

[56] Gao N, Chen YZ, Chi FL, Zhang TY, Xu HD, Kang HY, Pan TZ (2013). The frequency response of a floating piezoelectric microphone for the implantable middle ear microphone. Laryngoscope.

[57] Czarny J. Conception, fabrication and characterization of a MEMS microphone. Ph.D. thesis, INSA de Lyon. 2015. https://tel.archives-ouvertes.fr/tel-01247487.

[58] Czarny J, Walther A, Desloges B, Robert P, Redon E, Verdot T, Ege K, Guianvarc’h C, Guyader JL. New architecture of MEMS microphone for enhanced performances. In: Semiconductor conference Dresden–Grenoble (ISCDG), 2013 International. IEEE. 2013. p. 1–4.

[59] Wiskerke P, Havranek M. Implantable microphone system and calibration process. Google Patents. US Patent App. 13/124,244. 2009.

[60] Wiskerke P, Bervoets W. Implantable microphone for an implantable hearing prothesis. Google Patents. US Patent 8,200,339. 2012.

[61] Woo S-T, Jung E-S, Lim H-G, Lee Y-J, Seong K-W, Lee J-H, Cho J-H (2012). The design of temporal bone type implantable microphone for reduction of the vibrational noise due to masticatory movement. J Sens Sci Technol.

[62] Kotzar G, Freas M, Abel P, Fleischman A, Roy S, Zorman C, Moran JM, Melzak J (2002). Evaluation of MEMS materials of construction for implantable medical devices. Biomaterials.

[63] Ko WH (2007). Trends and frontiers of MEMS. Sens Actuators A Phys.

[64] Deterre M. MEMS integration for smart medical devices: opportunities and challenges. In: Design, test, integration and packaging of MEMS/MOEMS (DTIP), 2012 symposium on. IEEE. 2012. p. 253–7.

[65] Knisely K, Grosh K. A MEMS AlN transducer array for use as a cochlear implant. In: Applications of ferroelectric and workshop on the piezoresponse force microscopy (ISAF/PFM), 2013 IEEE international symposium on the. IEEE. 2013. p. 240–3.

[66] Safari A, Akdogan EK (2008). Piezoelectric and acoustic materials for transducer applications.

[67] Kaajakari V (2009). Practical MEMS.

[68] Acar C, Shkel AM (2003). Experimental evaluation and comparative analysis of commercial variable-capacitance MEMS accelerometers. J Micromech Microeng.

[69] Bell DJ, Lu T, Fleck NA, Spearing SM (2005). Mems actuators and sensors: observations on their performance and selection for purpose. J Micromech Microeng.

[70] Wilmott D, Alves F, Karunasiri G (2016). Bio-inspired miniature direction finding acoustic sensor. Sci Rep.

[71] Tadigadapa S, Mateti K (2009). Piezoelectric MEMS sensors: state-of-the-art and perspectives. Meas Sci Technol.

[72] Pfiffner F, Prochazka L, Péus D, Dobrev I, Dalbert A, Sim JH, Kesterke R, Walraevens J, Harris F, Röösli C (2017). A MEMS condenser microphone-based intracochlear acoustic receiver. IEEE Trans Biomed Eng.

[73] Jia XH, Gao N, Xu XD, Wu YZ, Kang HY, Chi FL (2016). A new floating piezoelectric microphone for the implantable middle ear microphone in experimental studies. Acta Oto-Laryngol.

[74] O’Connor KN, Puria S (2008). Middle-ear circuit model parameters based on a population of human ears. J Acoust Soci Am.

[75] Note L.P.T. Capacitive sensor operation and optimization. LT03-0020. vol. 5. 2009.

[76] Instruments T. Amplifiers for 3 wire analog electret microphones. Technical report, LMV1032-06/LMV1032-15/LMV1032-25 Datasheet. 2013.

[77] Sirohi J (2000). Fundamental understanding of piezoelectric strain sensors. J Intell Mater Syst Struct.

